# Single‐Molecule Observation of Intermediates in Bioorthogonal 2‐Cyanobenzothiazole Chemistry

**DOI:** 10.1002/anie.202005729

**Published:** 2020-08-11

**Authors:** Yujia Qing, Mira D. Liu, Denis Hartmann, Linna Zhou, William J. Ramsay, Hagan Bayley

**Affiliations:** ^1^ Department of Chemistry University of Oxford Oxford OX1 3TA UK; ^2^ W. M. Keck Science Department Claremont McKenna College Claremont CA 91711 USA; ^3^ Present address: Department of Chemistry University of California Berkeley CA 94720-1460 USA

**Keywords:** bioorthogonal chemistry, click chemistry, nanoreactors, single-molecule studies, tetrahedral intermediates

## Abstract

We report a single‐molecule mechanistic investigation into 2‐cyanobenzothiazole (CBT) chemistry within a protein nanoreactor. When simple thiols reacted reversibly with CBT, the thioimidate monoadduct was approximately 80‐fold longer‐lived than the tetrahedral bisadduct, with important implications for the design of molecular walkers. Irreversible condensation between CBT derivatives and N‐terminal cysteine residues has been established as a biocompatible reaction for site‐selective biomolecular labeling and imaging. During the reaction between CBT and aminothiols, we resolved two transient intermediates, the thioimidate and the cyclic precursor of the thiazoline product, and determined the rate constants associated with the stepwise condensation, thereby providing critical information for a variety of applications, including the covalent inhibition of protein targets and dynamic combinatorial chemistry.

## Graphical abstract

**Better off alone**: A nanoreactor approach was used to investigate the chemistry of2‐cyanobenzothiazole (CBT), an important bioorthogonal reagent, at the single‐molecule level. Reactions with thiols and aminothiols revealed mechanistic and kinetic information with important implications for several essential applications of CBT reagents.

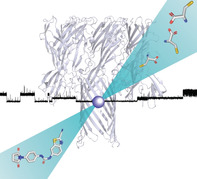

## Introduction

Orthogonal chemistry that proceeds rapidly at mild temperatures in aqueous solution is desired for the chemical manipulation of biological systems. A valuable example is condensation between 1,2‐aminothiols and 2‐cyanobenzothiazole (CBT), a reaction that occurs naturally in the synthesis of firefly luciferin.^1^ The reaction has been established as a biocompatible click strategy for targeting engineered N‐terminal cysteine residues with a rate of 3 to 9 m ^−1^ s^−1^ under physiological conditions.^2^, ^3^ Applications of this chemistry include site‐selective labeling of biomolecules,^2^, ^4^, ^5^, ^6^ and in situ assembly of nanostructures for molecular imaging in vitro and in vivo.^7^, ^8^, ^9^, ^10^, ^11^

**Figure 1:**
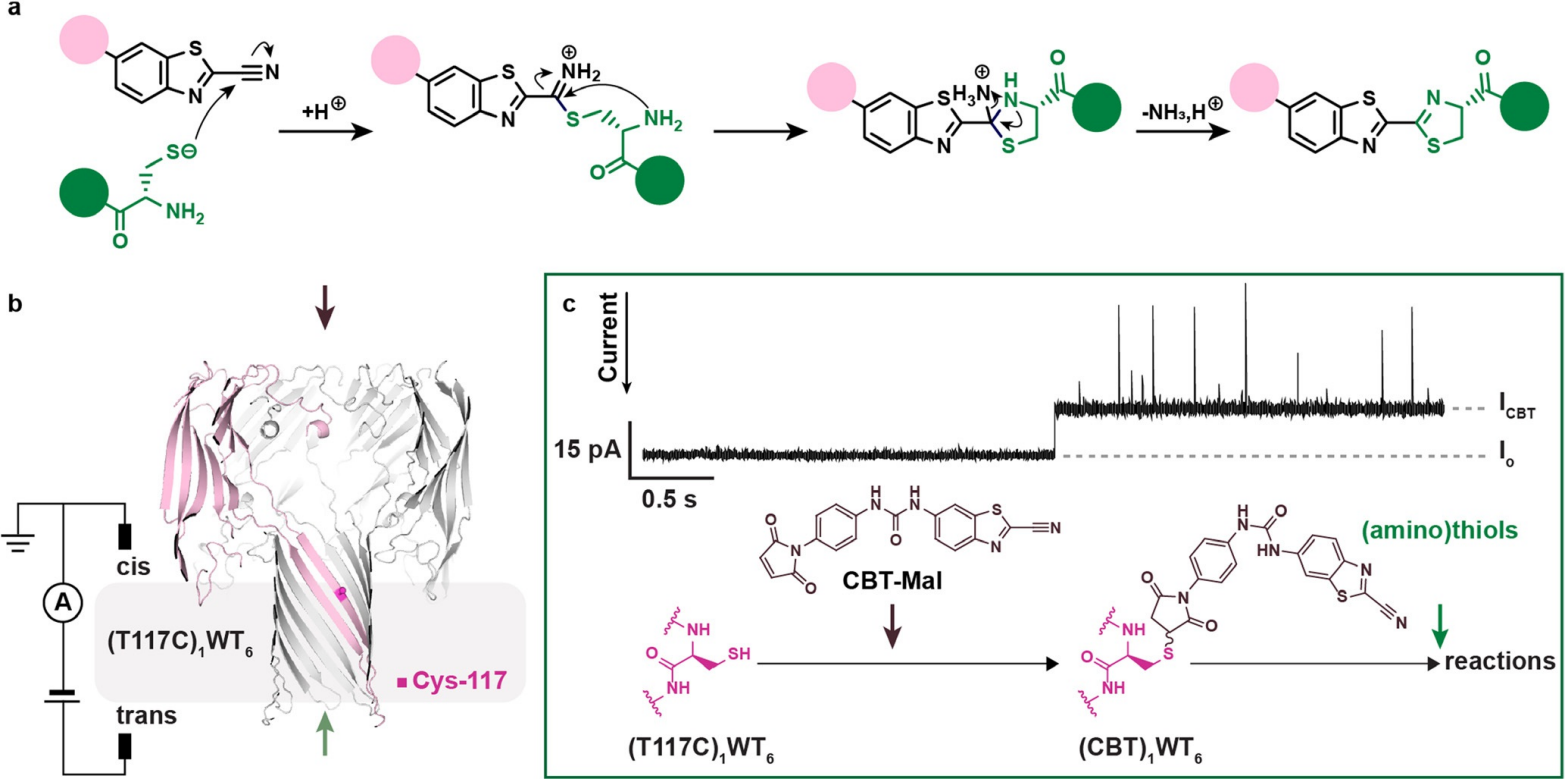
Single‐molecule 2‐cyanobenzothiazole (CBT) chemistry. a) Proposed mechanism for CBT click chemistry. b) Experimental setup and structure of the αHL heteroheptamer used in this work, which contained a single cysteine residue (magenta) at position 117 in one of the seven subunits. c) In situ generation of a CBT nanoreactor. After a single αHL heptamer had been inserted into a lipid bilayer to give stable open pore current (*I*_o_), CBT‐Mal was added from the *cis* side (10 μm). A step decrease in current (*I*_res%_=*I*_CBT_/*I*_o_=85.4±1.6 %, *n*=50) with increased noise (I_RMS(o)_=0.56±0.15 pA, *n*=50; I_RMS(CBT)_=0.88±0.13 pA, *n*=50) indicated irreversible modification of Cys‐117 with CBT‐Mal through Michael addition. The CBT nanoreactor was further reacted with (amino)thiols introduced from the trans side. All measurements were conducted at −50 mV (*trans*). Current traces were filtered at 1 kHz. Conditions: 2 m KCl, 20 mm HEPBS, pH 8.0, 20 μm EDTA, −50 mV, 20±1 °C.

A simple three‐step mechanism has been proposed for the reaction of CBT with aminothiols (Figure 1 a): 1) the thiolate attacks the nitrile carbon to form a thioimidate; 2) intramolecular attack of the terminal amine at the imino‐carbon generates a tetrahedral intermediate; 3) deamination of the tetrahedral species releases ammonia and a thiazoline as the final products.^5^ However, further detail is lacking. While the addition of monothiols to CBT is generally reversible,^2^ irreversible formation of a thioimidate has been reported on the internal cysteine residues within a bespoke peptide tag.^12^ In addition, it is not clear whether alternative elimination pathways exist, stemming from the tetrahedral intermediate. Amidines, the products of thiolate expulsion, have been detected after CBT treatment of peptide sequences containing either N‐terminal cysteine^13^ or internal cysteine and lysine residues.^14^ Reversion from the tetrahedral intermediate back to the thioimidate through ring opening has not been reported.

Capture of the intermediates, which is critical for validation of the mechanism and kinetic analysis of the reaction steps, has proved challenging for ensemble techniques. Previous employment of a microreactor coupled with induced nanoelectrospray ionization mass spectrometry (MS) caught and identified a tetrahedral intermediate for the reaction between CBT and l‐cysteine based on its fragmentation pattern, whereas the thioimidate remained elusive.^5^ To further the mechanistic understanding of this important reaction, we took a single‐molecule approach by using a protein nanoreactor. Specifically, the lumen of the protein nanopore, α‐hemolysin (αHL), was monofunctionalized with a CBT (Figure 1 b c). Individual bond‐making and bond‐breaking events within the nanoreactor caused characteristic time‐dependent changes in ionic current flow under an applied potential.^15^ The nanoreactor approach is ideal for studying CBT chemistry not only because of the high molecular sensitivity and sub‐millisecond temporal resolution,^16^ but also because of the biocompatible reaction conditions, which resemble the environment that prevails for actual applications of CBT reagents. By using this approach, we have, for example, differentiated photochemically and thermally interconverting isomers^17^, ^18^ and characterized a transient tetrahedral intermediate.^19^

## Results and Discussion

The CBT nanoreactors were built from individual αHL nanopores containing an inward‐facing cysteine at position 117 on one of the seven subunits (Figure 1 b). A single nanopore, inserted into a lipid bilayer, gave an open‐pore current (I_o_=86.8±5.3 pA, *n*=50) with root‐mean‐square noise (I_RMS_) of 0.56±0.15 pA under an applied potential of −50 mV (with respect to the trans compartment) after 1 kHz post‐recording filtering. When a maleimide derivative of CBT (CBT‐Mal, Figure 1 c) was introduced from the cis compartment, a partial current blockade occurred after a delay, indicating the formation of a CBT nanoreactor by covalent reaction with Cys‐117 (Figure 1 c). We functionalized the nanopore in situ at pH 8.0 to avoid nitrile hydrolysis,^5^, ^20^ which would occur during prolonged gel purification of modified pores under more basic conditions. The step decrease resulted in a residual current level (I_res%_=I_CBT_/I_o_) of 85.4±1.6 % (*n*=50) along with an increased I_RMS_ of 0.88±0.13 pA.

**Figure 2:**
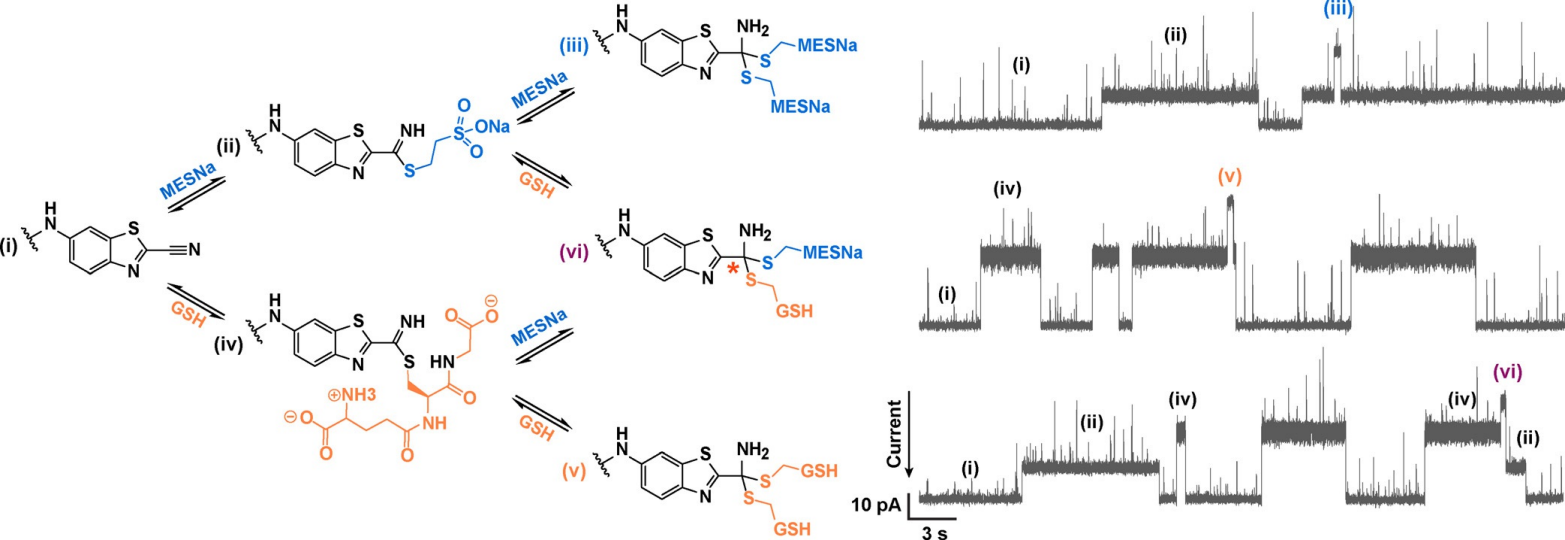
Reversible additions between the CBT and simple monothiols within the nanoreactor. Single‐channel recordings with the same pore showing the current associated with: (i) the CBT nanoreactor; (ii) the thioimidate formed after reaction with a single MESNa (*I*_res%_=71.7±1.6 %); (iii) the tetrahedral product formed with two MESNa molecules (*I*_res%_=53.2±1.3 %); (iv) the thioimidate formed after reaction with a single GSH (*I*_res%_=52.8±1.3 %); (v) the tetrahedral product formed with two GSH molecules (*I*_res%_=28.8±1.4 %); (vi) the tetrahedral product formed with one MESNa and one GSH molecule (*I*_res%_=39.7±1.3 %). The latter contains a newly generated chiral center (orange asterisk). MESNa (5 mm) and GSH (10 mm) were added to the trans compartment. Current traces were filtered at 200 Hz. Conditions: 2 m KCl, 50 mm HEPBS, pH 8.0, 20 μm EDTA, −50 mV (trans), 20±1 °C.

**Figure 3:**
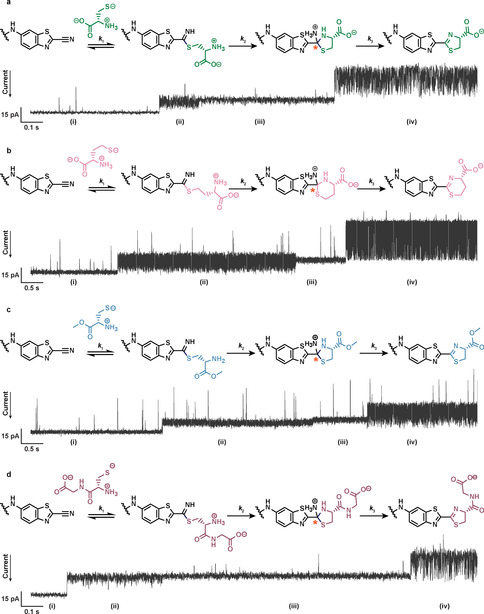
Irreversible condensations between individual CBT nanoreactors and aminothiols. Single‐channel recordings showing the three‐step reactions with a) Cys, b) hCys, c) CysOMe, and d) Cys‐Gly. Current levels correspond to the: (i) CBT nanoreactor; (ii) thioimidate; (iii) tetrahedral intermediate (containing a newly generated chiral center, orange asterisk); (iv) thiazoline or dihydrothiazine product. Aminothiols (5 mm) were added to the trans compartment. Current traces were filtered at 1 kHz. Conditions: 2 m KCl, 20 mm HEPBS, pH 8.0, 20 μm EDTA, −50 mV, 20±1 °C.

To monitor CBT chemistry in real‐time, reactants were added to the trans compartment. Reversible formation of adducts was seen with monothiols, including sodium 2‐mercaptoethanesulfonate (MESNa), glutathione (GSH) (Figure 2), N‐acetyl‐l‐cysteine (NAcCys), and 2‐mercaptoethanol (2ME; Figure S1 in the Supporting Information). Three‐step irreversible condensations were observed with aminothiols: l‐cysteine (Cys), l‐homocysteine (hCys), l‐cysteine methyl ester (CysOMe), and the dipeptide (Cys‐Gly), in keeping with the proposed reaction mechanism (Figure 3). These reactions did not occur when a non‐functionalized nanopore was tested with simple monothiols or aminothiols.

First, reversible reactions between various monothiols and CBT were characterized. The addition of MESNa to the trans compartment resulted in two new current levels (Figure 2; I_MESNa1_ and I_MESNa2_) with I_res%_ of 71.7±1.6 % and 53.2±1.3 % (*n*=3), respectively. The noise level was the highest for I_MESNa1_ and lowest for I_CBT_ (i.e. I_RMS_ ratio I_CBT_/I_MESNa1_I_MESNa2_=1.0:3.6:1.9). Given that transitions were only seen between I_MESNa1_ and I_CBT_ or I_MESNa1_ and I_MESNa2_, we assigned the first level as the thioimidate and the second level as the tetrahedral species formed with two MESNa molecules (Figure 2), which was further supported by kinetic analysis.

We created a three‐state model (Figure S2a) to determine the rate constants for the observed transitions (Figure 2) using QuB software.^21^ As expected for a bimolecular step, the reciprocal of the mean inter‐event interval (⟨*τ_i_*⟩) for the first step was proportional to the monothiol concentration (Figure S2b), and yielded a rate constant for thioimidate formation (*k*_1_) at pH 8.0 of 1.2±0.1 m ^−1^ s^−1^ by using *k*_1_=1/(⟨*τ_i_*⟩[RSH]), where [RSH] is the MESNa concentration. In contrast, the reciprocal of the mean waiting time before the transition from I_MESNA1_ back to I_CBT_ (⟨*τ*_−1_⟩) was independent of MESNa concentration (Figure S2c), in line with a unimolecular dissociation where *k*_−1_=1/⟨*τ*_−1_⟩=0.030±0.002 s^−1^. Similarly, rate constants were derived for the reversible formation of the tetrahedral adduct with a second MESNa molecule (*k*_2_=0.18±0.02 m ^−1^ s^−1^, *k*_−2_=2.4±0.5 s^−1^; Figure S2).

When two distinct monothiols were used (MESNa and GSH), the three possible tetrahedral adducts were distinguished by their current levels (Figure 2). In this case, tetrahedral adducts were rarely formed with two GSH molecules or with one MESNa and one GSH molecule, presumably due to steric hindrance. The enantiomers potentially formed with the mixed thiols were not detected in this experiment, presumably because the newly formed chiral center was distant from the chiral environment of the protein wall. Using conventional ESI‐MS, we failed to detect these tetrahedral adducts alongside the thioimidates, presumably because the steady‐state concentrations were too low (e.g. for MESNa, *K*_d1_=*k*_−1_/*k*_1_=0.025 m versus *K*_d2_=*k*_−2_/*k*_2_=13 m). Because the lifetime of the adduct with one ligand is over 80‐times longer than that with two ligands, CBT molecules have the capacity to act as molecular walkers on a multi‐thiol track,^22^ a possibility yet to be tested. It is worth noting that the termination of the reversible chemistry occurred always from the thioimidate level, presumably through hydrolysis.

We next examined stepwise irreversible condensations between aminothiols and CBT. Two intermediate states were resolved, which differed slightly in current amplitude (ΔI_res%_<5 %) but more markedly in I_RMS_ (Figure 3). This suggested an intramolecular transformation between two species of similar molecular mass but distinct conformational mobility, as opposed to the bimolecular additions, which caused significant current change (e.g., second addition of MESNa to CBT, ΔI_res%_=I_MESNa2 %_−I_MESNa1 %_≈20 %). Although the noise levels of the intermediates varied from pore to pore, I_RMS_ was always greater for the first intermediate than the second intermediate within a particular nanoreactor. The quotient of I_RMS_ values for the first and second intermediates (I_RMS1_/I_RMS2_) was 1.8±0.4 for Cys (*n*=20), 2.3±1.0 for hCys (*n*=20), 1.4±0.1 for CysOMe (*n*=5), and 1.5±0.3 for Cys‐Gly (*n*=6). The thiazoline products formed with Cys, CysOMe, and Cys‐Gly, or the dihydrothiazine product formed with hCys all exhibited an unusual “noisy” appearance, which arose from rapid interconversions between two discrete levels (Figure 3, Figures S3 and S4). The amplitude differences between these two interchanging levels were 30.4±3.3 pA (*n*=20) for Cys, 32.3±7.0 pA (*n*=20) for hCys, 20.9±1.6 pA (*n*=5) for CysOMe, and 26.5±3.8 pA for Cys‐Gly (*n*=6). When the reaction site was moved down the β barrel, from position 117 to position 121 or 123, the noise difference between the intermediates formed with Cys was diminished, but the current pattern of interconverting levels remained for the thiazoline product (Figure S5). To confirm the identity of the product formed with CysOMe, we synthesized the maleimide derivative of amino‐luciferin methyl ester and appended the product directly onto Cys‐117 in a nanopore (see the Supporting Information). The resulting construct replicated the “noisy” appearance of the product formed in situ (Figure S6). We attributed the current signature of the product to its conformational flexibility (Figure S7), which allows extensive interactions with the nanopore interior (see Section S3.1 in the Supporting Information for further discussion). Considering all these observations, we assigned the first intermediate as the thioimidate, the second as the tetrahedral intermediate formed by intramolecular cyclization, and the product as the thiazoline for Cys, CysOMe, and Cys‐Gly, or the dihydrothiazine for hCys. We assigned the protonation state of each species (Figure 3) based on knowledge of p*K*_a_ values (Table 1) and the energy levels previously computed for the protonation states of the intermediates formed during the reaction between CBT and Cys.^23^

**Table 1:** Rate constants for reactions between a CBT nanoreactor and (amino)thiols.

	p*K*_a_ (RSH)^[a]^	p*K*_a_ (RNH_3_^+^)^[a]^	*k*_1_ [m ^−1^ s^−1^]^[b]^	*k*_2_ [s^−1^]^[b]^	*k*_3_ [s^−1^]^[b]^	*n*
Cys	8.2, 8.3, 8.4	10.4, 10.8	1.0±0.2	5.3±1.2	0.21±0.05	20
hCys	8.7, 8.9, 9.9	10.5, 10.9	0.60±0.12	0.13±0.03	0.13±0.03	20
CysOMe	6.6, 7.0	9.0	5.2±2.3	0.036±0016	0.59±0.31	5
Cys‐Gly	6.4	9.6	0.44±0.23	5.6±2.4	0.88±0.42	6

	p*K*_a_ (RSH)^[a]^	*k*_1_ [m ^−1^ s^−1^]^[b]^	*k*_−1_ [s^−1^]^[b]^	*k*_2_ [m ^−1^ s^−1^]^[b]^	*k*_−2_ [s^−1^]^[b]^	
MESNa	9.2	1.2±0.1	0.030±0.002	0.18±0.02	2.4±0.5	3
GSH^[c]^	8.6, 8.8, 9.1	3.1±0.1	0.16±0.02	/^[d]^	/^[d]^	3

[a] Data for p*K*_a_ values are taken from references 25, 40, 41, 42, 43. Different p*K*_a_ values for the same compound from different studies are included. [b] Dwell‐time analysis and rate constant estimations were performed by using the maximum interval likelihood algorithm of QuB software. [c] Intramolecular attack by the internal amine in GSH to the thioimidate was not observed, in line with the unlikely formation of a 9‐membered ring. [d] Rate constants were not determined due to insufficient events.

For the three‐step condensations of CBT with Cys, hCys, CysOMe, and Cys‐Gly, we recorded individual lifetimes for the unreacted CBT nanoreactor, the thioimidate, and the tetrahedral intermediate (*τ_i_*, *τ_ii_*, *τ_iii_*). We created a four‐state model to derive the kinetic rate constant for each step by using the hidden Markov modeling within QuB software.^21^ Out of more than 50 irreversible condensations with Cys, hCys, CysOMe, and Cys‐Gly, we observed only twice that the thioimidate reverted back to CBT (Figure S8), in both cases with hCys. Hence, for CBT reactions with Cys, CysOMe, and Cys‐Gly, we set all three steps in the model as irreversible. In the case of hCys, we set the thioimidate formation as reversible and the later two steps as irreversible.

For step (i), in which the thiolate attacks the CBT nitrile, we calculated the second‐order rate constant (*k*_1_) for each aminothiol by using *k*_1_=1/(⟨*τ*_i_⟩[RSH]), where ⟨*τ*_i_⟩ is the mean lifetime of the unreacted CBT nanoreactor, and [RSH]=5 mm, the aminothiol concentration (Table 1). At pH 8.0, the values of *k*_1_ spanned 0.5 to 5 m ^−1^ s^−1^. *k*_1_(Cys) (1.0±0.2 m ^−1^ s^−1^) and *k*_1_(hCys) (0.60±0.12 m ^−1^ s^−1^) were around one order of magnitude smaller than the corresponding overall rates determined by spectroscopic methods.^3^, ^8^, ^24^ This was attributed to the need for the free reactant to attain the correct orientation for reaction within a confined space.^15^ The regeneration of CBT was not recorded during reactions with Cys, CysOMe, and Cys‐Gly, which indicated that the rate of the reverse reaction (*k*_−1_) was much smaller than the rate of the following step (*k*_2_) in these cases. Our observations with hCys confirmed the reversibility of the first step, in which the mean lifetime time of the thioimidate was governed by ⟨*τ*_ii_⟩=1/(*k*_−1_+*k*_2_)=⟨*τ*_−1_⟩⟨*τ*_2_⟩/(⟨*τ*_−1_⟩ + ⟨*τ*_2_⟩). Hence, we determined *k*_−1_ to be 0.012±0.009 s^−1^ for hCys and estimated *k*_−1_ to be less than *k*_2_/*n* (*n*=number of repeats) for the other cases where no reversal was observed.

For step (ii), in which the terminal amine attacks the thioimidate, the first‐order rate constant (*k*_2_) is the inverse of the mean waiting time before cyclization: *k*_2_=1/⟨*τ*_ii_⟩ for Cys, CysOMe, and Cys‐Gly; *k*_2_=1/⟨*τ*_2_⟩ for hCys (Table 1). Interestingly, the Cys derivative cyclized to the tetrahedral intermediate around 40‐times more quickly (*k*_2_=5.3±1.2 s^−1^) than the hCys derivative (*k*_2_=0.13±0.03 s^−1^). For the terminal amines associated with the thioimidates formed with Cys and hCys, we expect similar basicity (RNH_3_^+^: p*K*_a_≈9.0), given the similar p*K*_a_ values for the α‐amino groups in the aminothiols (RNH_3_^+^: p*K*_a_=10.8 for Cys and 10.9 for hCys^25^) or S‐methylated aminothiols (RNH_3_^+^: p*K*_a_=8.8 for S‐methyl‐l‐cysteine and 9.1 for l‐methionine^26^, ^27^). Therefore, the effective concentration of the nucleophile must dominate to account for the rate difference, which is consistent with the intramolecular cyclization rates of bifunctional compounds, where 6‐membered rings are formed approximately 30–100 times more slowly than 5‐membered rings.^28^, ^29^, ^30^, ^31^ The dipeptide (Cys‐Gly) had a similar rate of cyclization to Cys, while the methyl ester derivative of cysteine (CysOMe) cyclized approximately 150 times more slowly. With a p*K*_a_ value of around 7,^32^, ^33^ the thioimidate imine was unlikely to be mostly protonated at pH 8.0, which was a prerequisite for nucleophilic attack at a thioimidate carbon during thioimidate hydrolysis.^33^ Previous computational studies suggested intramolecular proton transfer from the protonated terminal amine to the neutral imine for the reaction between CBT and Cys.^23^ Given that S‐methyl‐l‐cysteine methyl ester has a p*K*_a_ of 6.9,^34^ we expect the terminal amine in the thioimidate formed with CysOMe to be mostly in the neutral form at pH 8.0. In contrast, the terminal amine in the cases of Cys, hCys, or Cys‐Gly (estimated p*K*_a_≈9.0) was likely protonated. Therefore, the much slower rate of cyclization with CysOMe might be caused by decreased proton availability from the terminal amine.

For the deamination step (iii), the *k*_3_=1/⟨*τ*_iii_⟩ values showed that the rates of elimination across different aminothiols were of the same magnitude (0.13 to 0.88 s^−1^). In comparison with the thioimidates preceding them, the lifetimes of the tetrahedral intermediates were much longer in the cases of Cys (⟨*τ*_iii_⟩ versus ⟨*τ*_ii_⟩: 4.8 s versus 0.19 s) and Cys‐Gly (1.1 s versus 0.18 s), shorter for CysOMe (1.7 s versus 28 s), and comparable for hCys (7.6 s versus 3.7 s). Indeed, only the relatively longer‐lived tetrahedral intermediate in the CBT reaction with Cys was intercepted in the previous MS study.^5^

To probe whether the reaction pathway could diverge at the tetrahedral intermediate, we tested N‐methyl‐l‐cysteine (NMeCys) with the CBT nanoreactor. Upon reaction with CBT, four states were formed with distinct current amplitudes and noise patterns (Figure S9). Based on the observed transitions (Figure S9), the four states were tentatively assigned as the thioimidate, the tetrahedral intermediate with a protonated tertiary amine, the tetrahedral intermediate with a protonated primary amine and the amidine. Interestingly, the thioimidate was never regenerated from the tetrahedral species. An irreversible step occurred after rounds of transitions between the tetrahedral intermediates and the amidine, which led to two interconverting levels similar to those seen with Cys, hCys, CysOMe, and Cys‐Gly. While further experiments are needed to validate the proposed assignments for the CBT reaction with NMeCys, we can say that methylation of the α‐amino group of Cys produced a more complex reaction pathway with CBT than Cys.

## Conclusion

In conclusion, we have monitored CBT chemistry at the single‐molecule level in real‐time under biocompatible conditions. With simple monothiols, we observed the reversible formation of tetrahedral adducts on CBT with two thiol ligands, which might be applied to develop covalent inhibitors targeting multiple cysteines at the active sites of disease‐related proteins.^35^ We also envision that CBT chemistry will be a strong addition to the dynamic covalent chemistry toolbox and that the rate constants derived here will be key in achieving fine control of such systems.^36^ For example, a multi‐cysteine track can potentially interact with CBT molecules through consecutive dynamic covalent reactions, for which the association and dissociation kinetics determine the processivity of the mobile molecules.^22^, ^37^ When individual aminothiols reacted with a CBT nanoreactor, we were able to resolve for the first time two transient intermediate species in the formation of thiazolidines or dihydrothiazines based on the root‐mean‐square noise of their current states. Although the current noise associated with molecular structure cannot be predicted at present, our results will add to the data accumulating in this area.^38^, ^39^ The tracking of intermediates enabled stepwise kinetic analysis of various CBT reactions, which unveiled steps that were affected by the structure of the aminothiol. This might inform future design of self‐assembling materials based on CBT chemistry for applications in biology and medicine such as molecular imaging and therapeutic delivery. In addition to walker systems based on a multi‐cysteine track,^22^, ^37^ the formation of an amidine via the tetrahedral intermediate suggests the use of alternating cysteine and lysine footholds for CBT‐based walker systems. Therefore, we anticipate that the findings described here will inspire broad applications of CBT chemistry. Further, the approach we have developed might also be used to obtain additional mechanistic and kinetic information under various conditions including, importantly, low and high temperatures.

## Conflict of interest

The authors declare no conflict of interest.

## Supporting information

As a service to our authors and readers, this journal provides supporting information supplied by the authors. Such materials are peer reviewed and may be re‐organized for online delivery, but are not copy‐edited or typeset. Technical support issues arising from supporting information (other than missing files) should be addressed to the authors.

Supplementary
