# Supplementary material for: Single‐Molecule Observation of Intermediates in Bioorthogonal 2‐Cyanobenzothiazole Chemistry
Source: Angew Chem Int Ed Engl. 2020 Aug 11;59(36):15711–6. doi: 10.1002/anie.202005729 (PMC7496719; doi:10.1002/anie.202005729)
Supplement: Supplementary file 1 — Supplementary [file ANIE-59-15711-s001.pdf]

Angewandte Chemie
Eine Zeitschrift der Gesellschaft Deutscher Chemiker

Supporting Information

Single-Molecule Observation of Intermediates in Bioorthogonal 2-Cyanobenzothiazole Chemistry

*Yujia Qing, Mira D. Liu, Denis Hartmann, Linna Zhou, William J. Ramsay, and Hagan Bayley**

anie_202005729_sm_miscellaneous_information.pdf

Contents

1. Experimental 3

1.1 General 3

1.2 Protein preparation 3

1.3 Planar lipid bilayer recordings and data analysis 4

1.4 Synthesis of Mal-CBT 7

1.5 Synthesis of aLucOMe 7

1.6 Synthesis of Mal-LucOMe 8

2. Reversible reactions of monothiols with the CBT nanoreactor 9

3. Irreversible condensations of aminothiols with the CBT nanoreactor 12

3.1 Products of condensations between aminothiols and CBT nanoreactors 12

3.2 Direct attachment of the product molecule to a cysteine nanopore 17

3.4 Reversible thioimidate formation with hCys 20

3.5 Ireversible condensation with NMeCys 21

1. Experimental

1.1 General

Reagents were purchased from commercial sources (as indicated) and used as received. NMR spectra were recorded on a Bruker AVIII HD 400 spectrometer. Chemical shifts for ^{1}H are reported in ppm on the δ scale, and referenced to the residual solvent peak. Coupling constants (*J*) are reported in Hertz (Hz). The following abbreviations are used to describe signal multiplicity for ^{1}H spectra: s: singlet, d: doublet, t: triplet, m: multiplet, dd: doublet of doublets. Low and high resolution mass spectra were recorded on a Micromass Platform 1 spectrometer and a MicroTOF mass spectrometer using electrospray ionization.

1.2 Protein preparation

αHL monomers were prepared by in vitro transcription-translation (IVTT) as previously reported[1]. Specifically, engineered αHL polypeptides were expressed by using a commercial IVTT kit: E. coli T7 S30 Extract System for Circular DNA (Promega). To suppress transcription by E. coli RNA polymerase, the T7 S30 extract provided in the kit was treated with rifampicin prior to use (1 μg mL^{-1} final concentration). A standard reaction comprised: DNA template (3.2 μg), amino acid mix minus methionine (supplied with the kit, 5 μL), S30 premix without amino acids (supplied with the kit, 20 μL), [^{35}S]methionine (2 μL, 1,200 Ci $mmol^{-1}$, 15 mCi mL^{-1}, MP Biomedicals), T7 S30 extract (supplied with the kit, 15 μL), and nuclease-free water to a final volume of 50 μL. To make heteroheptamers, plasmids encoding the WT αHL and the mutant αHL with a D8 tag were mixed in a ratio 6:1 (WT: mutant). The IVTT mixture was incubated at 37°C for 1 h.

Heptamerization was carried out by the addition of rabbit erythrocyte membranes (3 μL, ~1 mg protein mL^{-1}) and incubation at 37°C for 1 h. The mixture was then centrifuged for 10 min at 25,000 × g. The supernatant was removed, and the pellet resuspended in MBSA buffer (200 μL, 10 mM 3-morpholinopropane-1-sulfonic acid (MOPS), 150 mM NaCl, 1 mg mL^{-1} bovine serum albumin, pH

7.4). The wash with MBSA was repeated before the pellet was resuspended in 2X Laemmli sample buffer (50 µL) and electrophoresed in a 5% SDS/PAGE gel at 70 V for 15 h. The αHL heptamers containing different numbers of mutant subunits were separated in the gel based on their different electrophoretic mobilities, which were determined by the number of octa-aspartate (D8) tails. The top and bottom bands corresponded to WT_7 and $(mutant\text{-}D8)_7$, respectively. The second band from the top was the desired heteroheptamer containing a single engineered subunit. To extract heptameric pores, the gel was first dried without fixation on Whatman 3M filter paper under vacuum for 5 h at room temperature. After visualization by autoradiography with Kodak BioMax MR film, the desired bands were cut out from the gel with a scalpel. Each excised band was rehydrated in TE buffer (300 µL, 10 mM Tris·HCl, 1 mM ethylenediaminetetraacetic acid (EDTA), pH 8.0) for 1 h at room temperature. The filter paper was then removed, and the rehydrated gel was macerated with a pestle. The resulting slurry was filtered through a 0.2 µm hydrophilic membrane filter (Proteus Mini Clarification Spin Column, Generon). The filtrate was stored in 10 µL aliquots at -80 °C.

1.3 Planar lipid bilayer recordings and data analysis

1,2-Diphytanoyl-sn-glycerol-3-phosphocholine (DPhPC) was purchased from Avanti Polar Lipids. Unless otherwise stated, the other chemicals were purchased from Sigma-Aldrich.

Planar bilayer recordings were performed following the method established by Montal and Mueller[2]. Two Delrin chambers were separated by a 25 µm-thick Teflon film containing an aperture (60 µm in diameter), which was pre-treated with 1% (v/v) hexadecane in pentane. Each chamber was then filled with buffer (500 µL, 2 M KCl, 20 mM N-(2-hydroxyethyl)piperazine-N'-(4-butanesulfonic acid) (HEPBS), 20 µM EDTA, pH 8.0). A drop of DPhPC in pentane (5 mg mL^{-1}) was added to both chambers. Repetitive up-and-down pipetting of the buffer solution resulted in the formation of a lipid bilayer across the aperture. A transbilayer potential was applied with two Ag/AgCl electrodes, each contained within salt bridges formed from 3 M KCl in 3% (w/v) low-melt agarose.

Ionic currents were recorded by using a patch clamp amplifier (Axopatch 200B, Axon Instruments) with a 4-pole low-pass Bessel filter (80 dB/decade) at room temperature (20 °C ± 1 °C). Signals were digitized with a Digidata 1320A digitizer (Molecular Devices), connected to a computer running the pCLAMP 9.2 software suite (Molecular Devices). Unless stated otherwise, the signal was filtered with a corner frequency of 10 kHz and sampled at 50 kHz.

To study CBT chemistry, gel-purified αHL heptamers containing a single cysteine were added to the grounded cis chamber. To facilitate pore insertion, a potential of +200 mV was applied with constant stirring in the cis compartment. After a single pore had inserted, the potential was changed to -50 mV and the stirring stopped. To build a CBT nanoreactor, CBT-Mal was added to the cis side as a freshly prepared DMSO solution followed by stirring for 30 s to give a final concentration of 10 μM and 0.2% DMSO in the cis compartment. Monothiols were prepared freshly in the recording buffer as 500 mM stock solutions and titrated with NaOH to pH 8.0 (recording buffer: 2 M KCl, 50 mM HEPBS, 20 μM EDTA, pH 8.0). Aminothiols were prepared freshly in the recording buffer as 250 mM stock solutions and titrated with NaOH to pH 8.0 (recording buffer: 2 M KCl, 20 mM HEPBS, 20 μM EDTA, pH 8.0). To initiate reactions with CBT, a reagent was mixed with 150 uL of recording solution taken from the trans compartment, which was added back to the trans compartment followed by stirring for 30 s. For the reversible reactions between CBT and monothiols that generated two current blockade levels, individual events were identified by using a threshold based single-channel search in Clampfit to generate idealized traces. The mean dwell times ($<\tau>$) of the CBT nanoreactor (i), the thioimidate (ii), and the tetrahedral adduct (iii) were determined using QuB software (QUB 2.0, Buffalo University). A maximum likelihood (MIL) algorithm was used to calculate the rate constants based on the idealized trace using a three-state model[3]. The bimolecular reactions between CBT and monothiols, and between thioimidates and monothiols were first order in monothiol concentration ($k = 1/(<\tau>[\text{monothiols}])$, $M^{-1}s^{-1}$), and the rate constants were extracted from the slopes of the plots ($1/<\tau>$ vs [monothiols]) determined by linear regression analysis. The dissociation of the

monothiols from either the tetrahedral adducts or the thioimidates was independent of the concentrations of monothiols, and the rate constants were obtained by averaging the dissociation rates ($k = 1/\langle\tau\rangle$, s^{-1}) obtained at different concentrations of monothiols.

For the irreversible condensations between CBT and aminothiols, current traces were idealized and imported into QuB as described above. A four-state model was used to derive the mean dwell times ($\langle\tau\rangle$) of the CBT nanoreactor (i), the thioimidate (ii), and the tetrahedral intermediate (iii) using MIL algorithm. The first step was bimolecular and hence followed $k = 1/\langle\tau\rangle$[aminothiol], whereas the later two steps were unimolecular and followed $k = 1/\langle\tau\rangle$. Once the thiazoline or dihydrothiazine was formed, a new experiment with a separate nanopore was started.

Transient spikes of varying amplitudes in the baseline of single-channel recordings were observed with the wild-type pore in the absence or presence of CBT-Mal (10 µM), with the cysteine mutant nanopore (e.g. 117C) before the reaction with CBT-Mal, and more frequently after the CBT nanoreactor was formed. Perfusion of the cis compartment three times after the functionalization to remove excess CBT-Mal did not alter the spiky character of the current. The transient spikes were therefore attributed to intrinsic fluctuations within the nanopore structure, which were more pronounced when the interior wall was modified. The spikes were neglected during analysis.

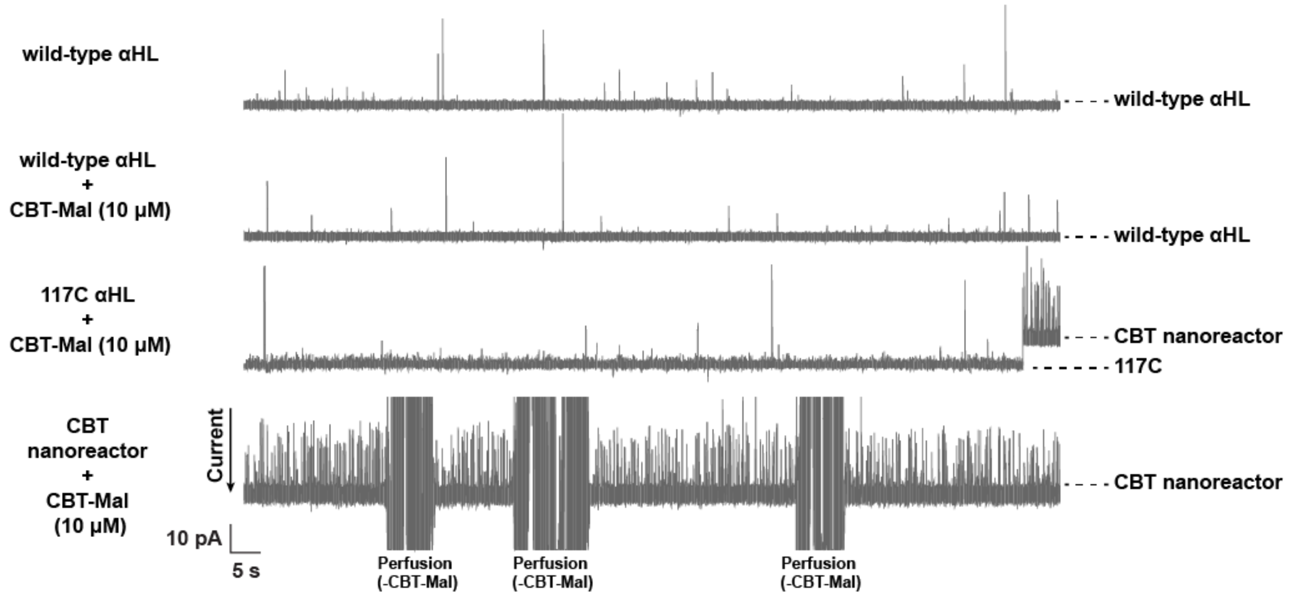

1.4 Synthesis of Mal-CBT

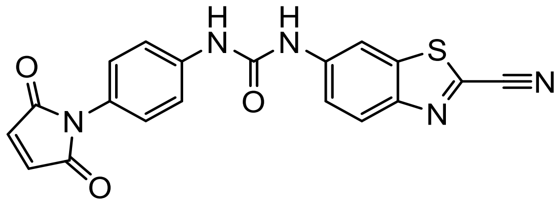

4-(Maleinimido)phenyl isocyanate (2.5 mg, 0.01mmol) and 2-cyano-6-aminobenzothiazole (5.1mg, 0.03 mmol) were dissolved in 100 µL anhydrous DMSO. The reaction mixture was shaken at room temperature for 2 h before purification by reverse phase HPLC to afford CBT-Mal (3.7 mg, 81.5%) as a pale yellow solid (Agilent 1260 Infinity; Supelcosil LC-18 column: 250 × 21.2 mm; linear gradient: 5- 95% eluant B in eluant A over 45 min, flow rate: 5 mL min^{-1}; eluant A: 0.1% TFA in water; eluant B: 0.1% TFA in acetonitrile). ^{1}H NMR (400 MHz, DMSO-d_6) δ ppm 7.17 (s, 2 H) 7.25 (d, *J* = 9.10 Hz, 2 H) 7.59 (d, *J* = 9.10 Hz, 2 H) 7.68 (dd, *J* = 9.05, 2.20 Hz, 1 H) 8.17 (d, *J* = 9.05 Hz, 1 H) 8.56 (d, *J* = 1.96 Hz, 1 H) 9.15 (s, 1 H) 9.43 (s, 1 H); m/z (ESI^+) ($[M+H]^+$) calculated 390.1, found 390.0.

1.5 Synthesis of aLucOMe

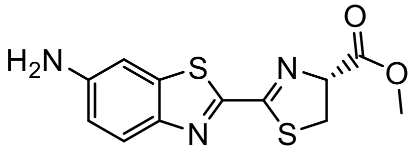

2-Cyano-6-aminobenzothiazole (11.6 mg, 0.07mmol) and L-cysteine methyl ester hydrochloride were dissolved in 200 µL DMSO, which then was supplemented with K_2CO_3 (2 mg) in 10 µL H_2O. The mixture was shaken at room temperature for 2 h and then centrifuged. The supernatant was purified by reverse phase HPLC to yield aLucOMe (11.0 mg, 56.8%) as a bright yellow solid (Agilent 1260 Infinity; Supelcosil LC-18 column: 250 × 21.2 mm; linear gradient: 5- 95% eluant B in eluant A over 45 min, flow rate: 5 mL min^{-1}; eluant A: 0.1% TFA in water; eluant B: 0.1% TFA in acetonitrile). ^{1}H NMR (400 MHz, acetonitrile-d_3) δ 3.72 (dd, *J* = 9.05, 5.40 Hz, 2 H) 3.80 (s, 3 H)

5.38 (t, *J* = 9.05 Hz, 1 H) 6.93 (dd, *J* = 8.80, 2.20 Hz, 1 H) 7.14 (d, *J* = 2.20 Hz, 1 H) 7.80 (d, *J* = 8.80 Hz, 1 H); m/z (ESI$^+$) ([M+H]$^+$) calculated 294.0, found 294.0.

1.6 Synthesis of Mal-LucOMe

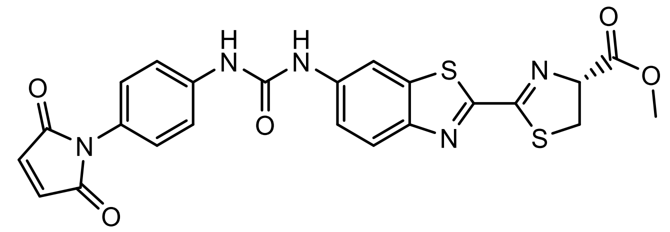

aLucOMe (1.3 mg, 4.67 μmol) and 4-(maleinimido)phenyl isocyanate (2.0 mg, 9.34 μmol) were dissolved in 100 μL anhydrous DMSO. The solution was shaken at room temperature for 2 h before purification of the product by reverse phase HPLC to afford Mal-LucOMe (2.1 mg, 92.8 %) as a pale yellow solid (Agilent 1260 Infinity; Supelcosil LC-18 column: 250 × 21.2 mm; linear gradient: 5- 95% eluant B in eluant A over 45 min, flow rate: 5 mL min^{-1}; eluant A: 0.1% TFA in water; eluant B: 0.1% TFA in acetonitrile). ^{1}H NMR (400 MHz, acetonitrile-d_3) δ 3.74 (dd, *J* = 9.29, 3.91 Hz, 2 H) 3.79 (s, 3 H) 5.39 (t, *J* = 9.17 Hz, 1 H) 6.90 (s, 2 H) 7.24 (d, *J* = 8.80 Hz, 2 H) 7.51 (dd, *J* = 9.05, 2.20 Hz, 1 H) 7.61 (d, *J* = 9.10 Hz, 2 H) 7.96 - 8.03 (m, 2 H) 8.19 (s, 1 H) 8.41 (d, *J* = 2.20 Hz, 1 H); m/z (ESI$^+$) 294.0 ([M+H]$^+$); HRMS (ESI$^+$) ([M+H]$^+$) calculated 508.07439, found 508.07454.

2. Reversible reactions of monothiols with the CBT nanoreactor

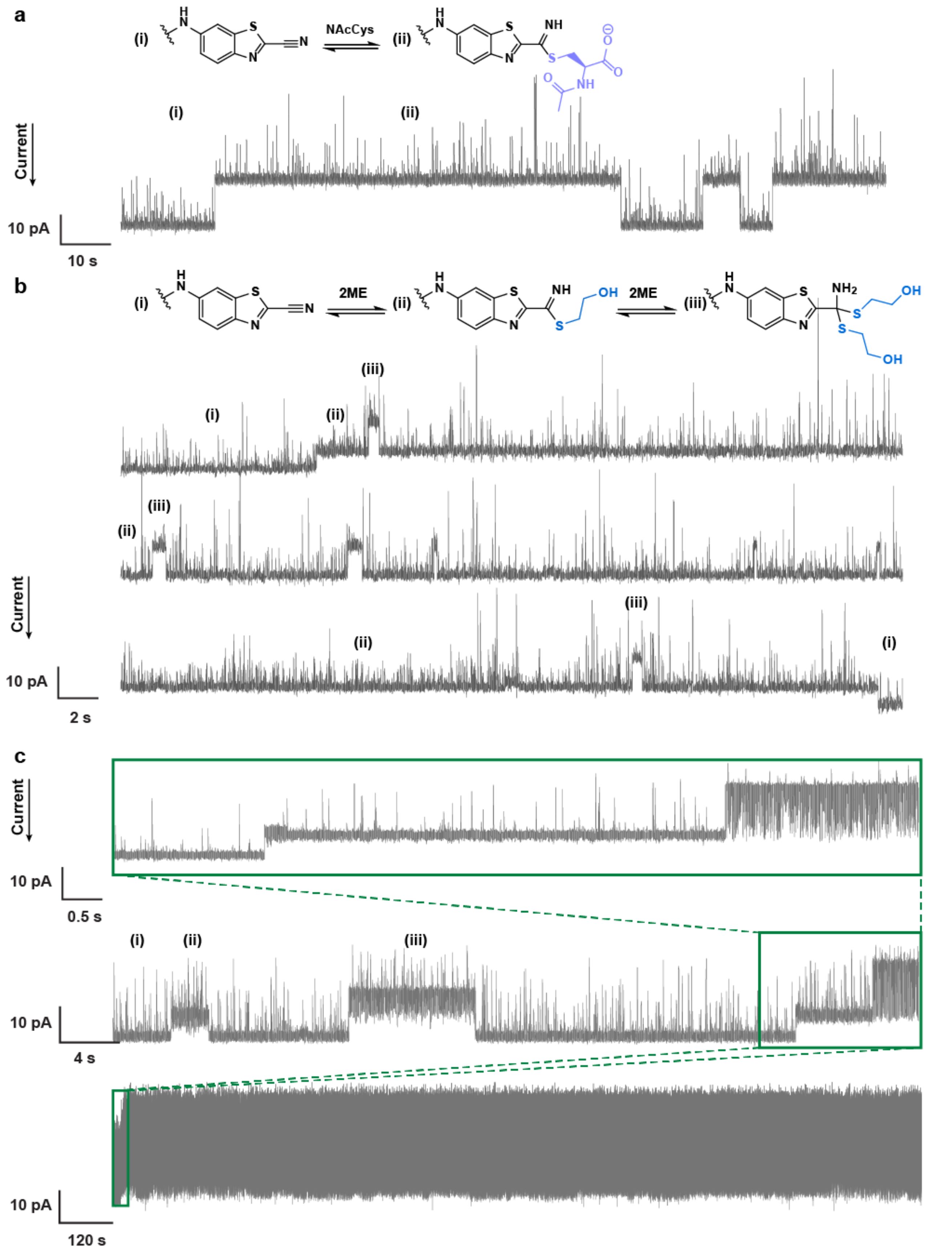

Figure S1. Reversible reactions of monothiols with the CBT nanoreactor. a) Single-channel recording of a CBT nanoreactor reacting with NAcCys (5 mM, trans) at −50 mV. The two current levels correspond to the CBT nanoreactor (i) and the thioimidate formed with one molecule of NAcCys (ii). Tetrahedral adducts were not observed within the short recording period (<20 min). A 200 Hz lowpass filter was applied post-recording. **b)** Single-channel recordings of a CBT nanoreactor reacting with 2ME (10 mM, trans) at -50mV. The three current levels correspond to the CBT nanoreactor (i), the thioimidate formed with one molecule of 2ME (ii), and the tetrahedral adduct formed with two molecules of 2ME (iii). A 200 Hz lowpass filter was applied post-recording. **c)** Single-channel recordings of a CBT nanoreactor reacting with MESNa (10 mM, trans), GSH (5 mM, trans), and Cys (5 mM, trans, added after MESNA and GSH). Reversible formation of thioimidates on the CBT nanoreactor (i) was observed with MESNa (ii) and GSH (iii), which was absent after the irreversible condensations with Cys (green box, the zoom in on the intermediates is shown in the top trace). The bottom trace was a 30 min recording after the condensations with Cys. A 1000 Hz lowpass filter was applied post-recording. Conditions: 2 M KCl, 50 mM HEPBS, pH 8.0, 20 μM EDTA, -50 mV, 20 ± 1 °C.

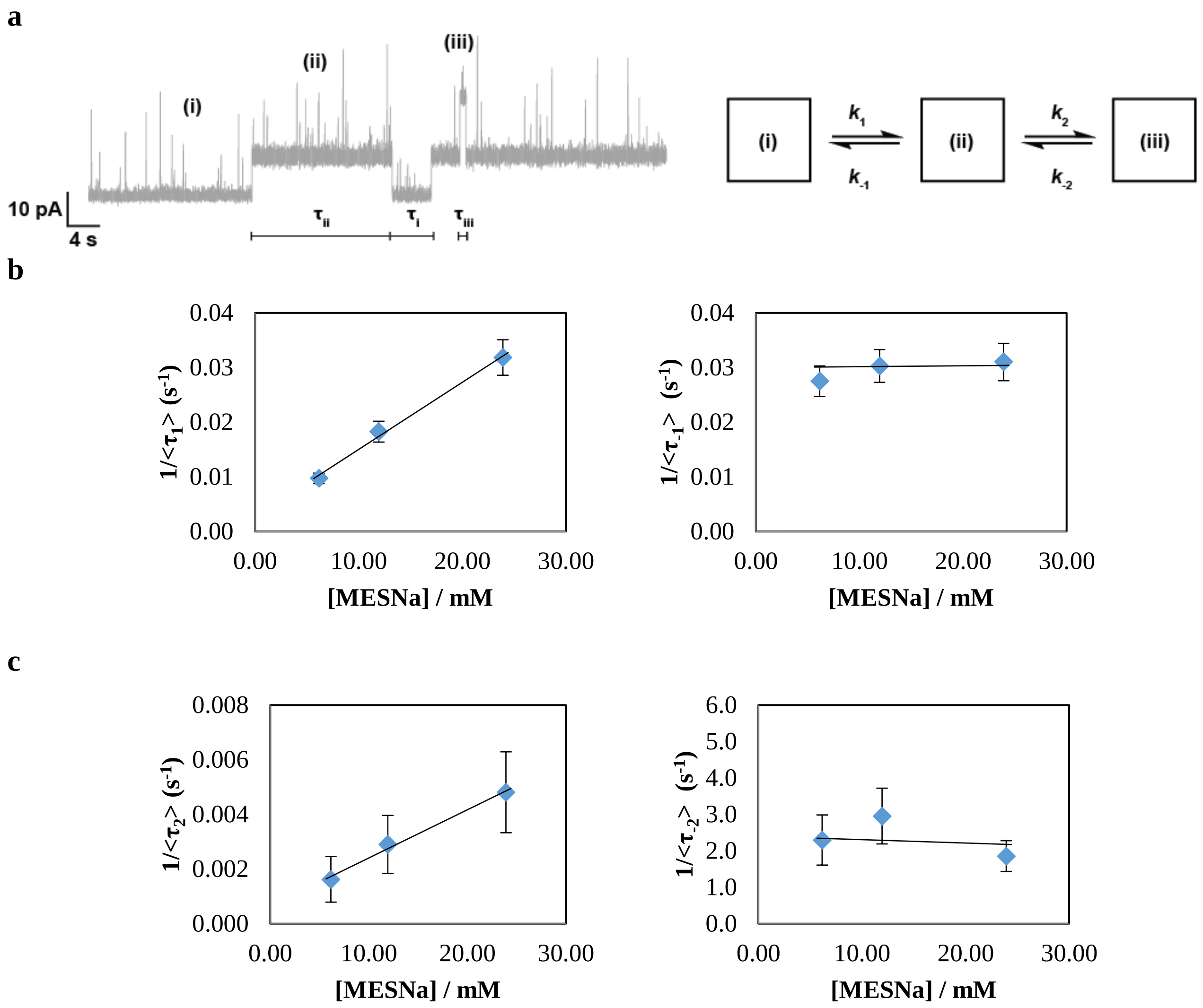

Figure S2. Concentration dependence of the reversible reactions between MESNa the CBT nanoreactors. a) The three-state model used to derive the rate constants for the observed transitions. **b)** Plots of the reciprocals of the mean inter-event intervals for the CBT nanoreactor ($<\tau_1>$) and the mean waiting times for the elimination of MESNa from the corresponding thioimidate ($<\tau_{-1}>$) versus the MESNa concentration. Calculated $k_1 = 1.2 \pm 0.1$ $M^{-1}s^{-1}$; averaged $k_{-1} = 0.030 \pm 0.002$ s^{-1}. **c)** Plots of the reciprocals of the mean waiting times for the formation of tetrahedral adducts with a second MESNa molecules ($<\tau_2>$) and the mean waiting times of regeneration of the thioimidate from the tetrahedral adduct ($<\tau_{-2}>$) versus the MESNa concentration. Calculated $k_2 = 0.18 \pm 0.02$ $M^{-1}s^{-1}$; averaged $k_{-2} = 2.4 \pm 0.5$ s^{-1}. $< \tau_{-1}>$ and $<\tau_2>$ were related to the mean lifetime of thioimidate ($<\tau_{ii}>$) based on $<\tau_{ii}> = 1/(k_{-1} + k_2) = <\tau_{-1}>< \tau_2>/(<\tau_{-1}> + < \tau_2>)$.

3. Irreversible condensations of aminothiols with the CBT nanoreactor

3.1 Products of condensations between aminothiols and CBT nanoreactors

Rapid interconversions between two discrete current levels were observed as the final product signature for all reactions between aminothiols and CBT nanoreactors. Different interconversion frequencies were recorded with different individual CBT nanoreactors upon reaction with the same aminothiol (e.g. Cys, Figure S3). With a given nanoreactor, the interconversion rate was highly voltage-dependent for charged molecules (e.g. the thiazoline formed with Cys or the dihydrothiazine formed with hCys) and less so with neutral species (e.g. the thiazoline formed with CysOMe) (Figure S4). We moved the tethering site of the reactive CBT group along the length of the β barrel to examine the change in product current pattern. At sites 117 and 121, interconversions between two states were seen after the reaction with Cys, whereas at position 123, interconversions were recorded between 4 states (Figure S5). We speculated that the appendant product molecule was able to interact with various sites on the interior wall.

In additions to the interactions with the inward-facing amino acid side chains, the tethered molecules might undergo internal conformational changes that contribute to the generation of current signatures of the products. For example, there might be cis-trans isomerization in the urea group through rotation about the C−N bond, for which the activation barrier has been reported to be 11.3 kcal mol^{-1} in DMF/DMSO solutions[4] (>15000 s^{-1} at 20 °C). There might also be cis-trans isomerization in the luciferin structure through rotation about the C-C bond connecting the thiazoline and the benzothiazole rings. While experimental measurements of the rotation rates are lacking, computational studies predicted energy barriers for rotations from the cis or trans conformations to be 1.7 and 7.2 kcal mol^{-1}, respectively[5].

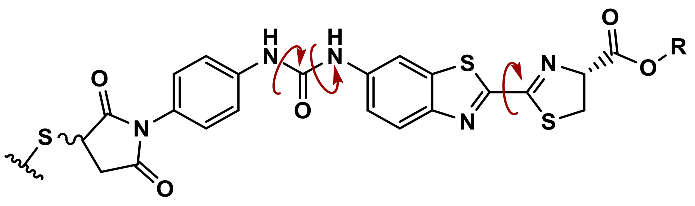

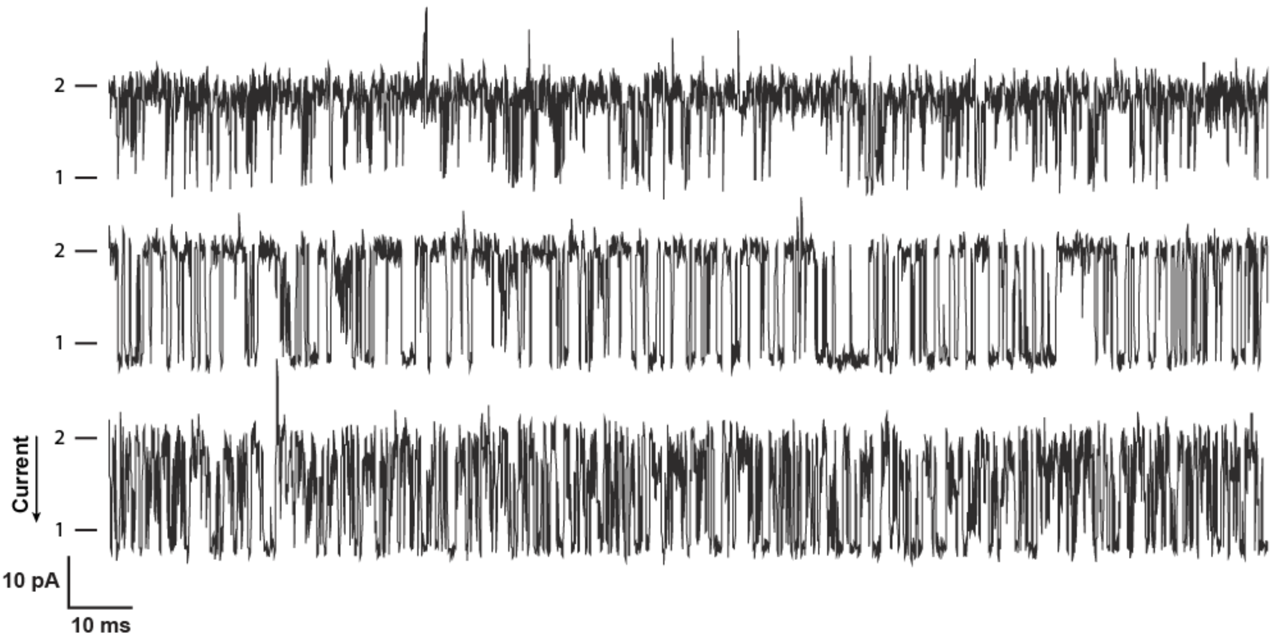

Figure S3. Single-channel recordings with three different CBT nanoreactors after reaction with Cys. The reaction products show two discrete states but each has a different interconversion rate (From top to bottom: $k_{1\text{-}2}$ = 8130 ± 40 s^{-1}, 3680 ± 20 s^{-1}, 2260 ± 100 s^{-1}; $k_{2\text{-}1}$ = 1220 ± 10 s^{-1}, 1260 ± 60 s^{-1}, 3060 ± 20 s^{-1}). No filter was applied post-recording to the current traces shown here. Cys (5 mM) was added to the trans compartment. Conditions: 2 M KCl, 20 mM HEPBS, pH 8.0, 20 μM EDTA, -50 mV, 20 ± 1 °C.

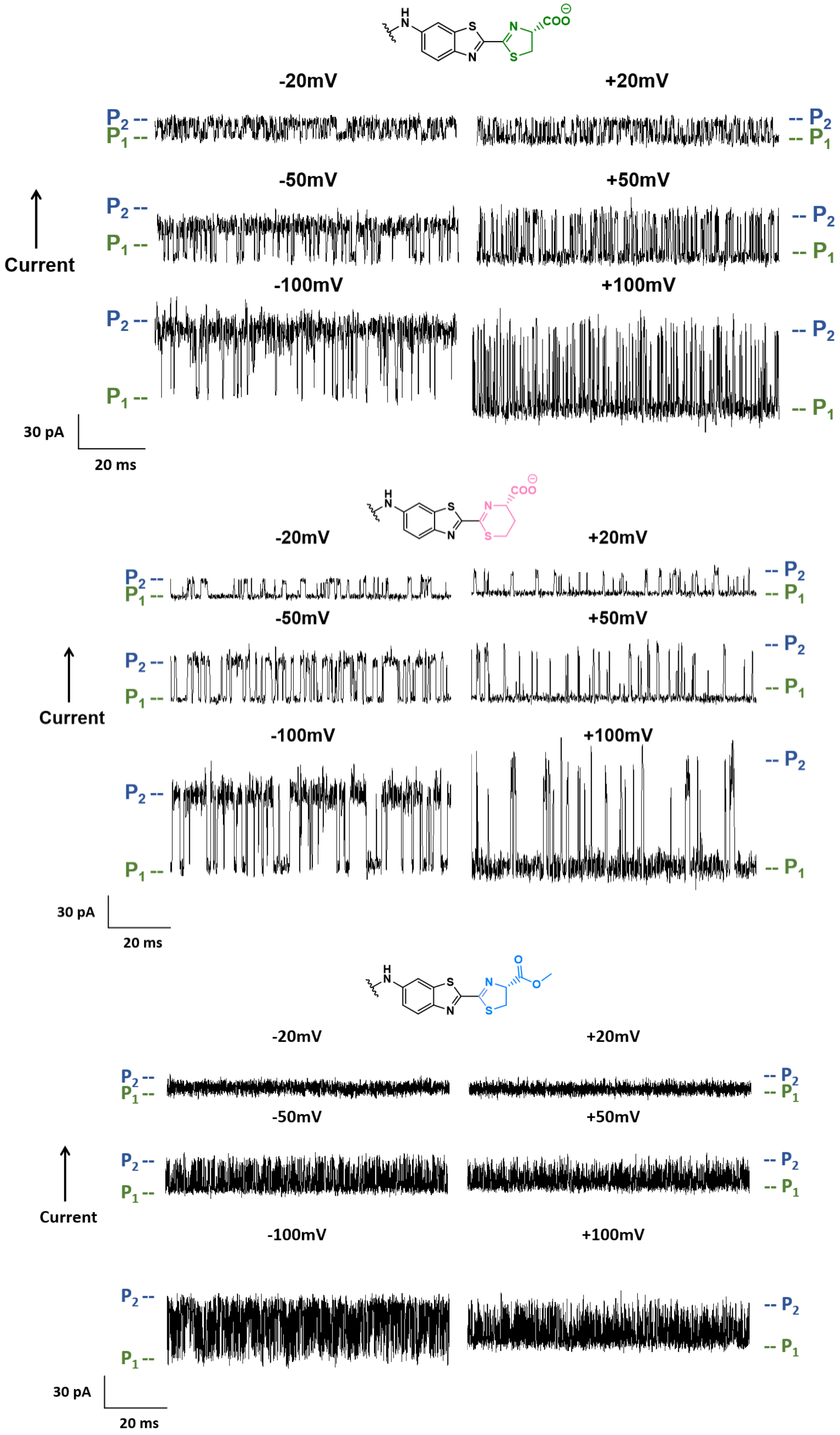

-20mV
+20mV
-50mV
+50mV
-100mV
+100mV
Current
30 pA
20 ms
COO
-20mV
+20mV
-50mV
+50mV
-100mV
+100mV
Current
30 pA
20 ms
-20mV
+20mV
-50mV
+50mV
-100mV
+100mV
Current
30 pA
20 ms

Figure S4. Representative current signatures of products formed with Cys, hCys, and CysOMe at different voltages. Rapid interconversions between two discrete current levels (P_1 and P_2) were observed for the thiazoline products formed with Cys and CysOMe, and the dihydrothiazine product formed with hCys. The rate of transition from P_1 to P_2 at -50 mV for the current traces shown: $k_{1\text{-}2}$ = 4380 ± 30 s^{-1} (Cys); 1010 ± 10 s^{-1} (hCys); 11200 ± 100 s^{-1} (CysOMe). The rate of transition from P_2 to P_1 at -50 mV for the current traces shown: $k_{2\text{-}1}$ = 1180 ± 10 s^{-1} (Cys); 1230 ± 10 s^{-1} (hCys); 20700 ± 100 s^{-1} (CysOMe). No filter was applied post-recording to the current traces shown here. Cys (5 mM), hCys (5 mM), or CysOMe (5 mM) was added to the trans compartment. Conditions: 2 M KCl, 20 mM HEPBS, pH 8.0, 20 µM EDTA, -50 mV, 20 ± 1 °C.

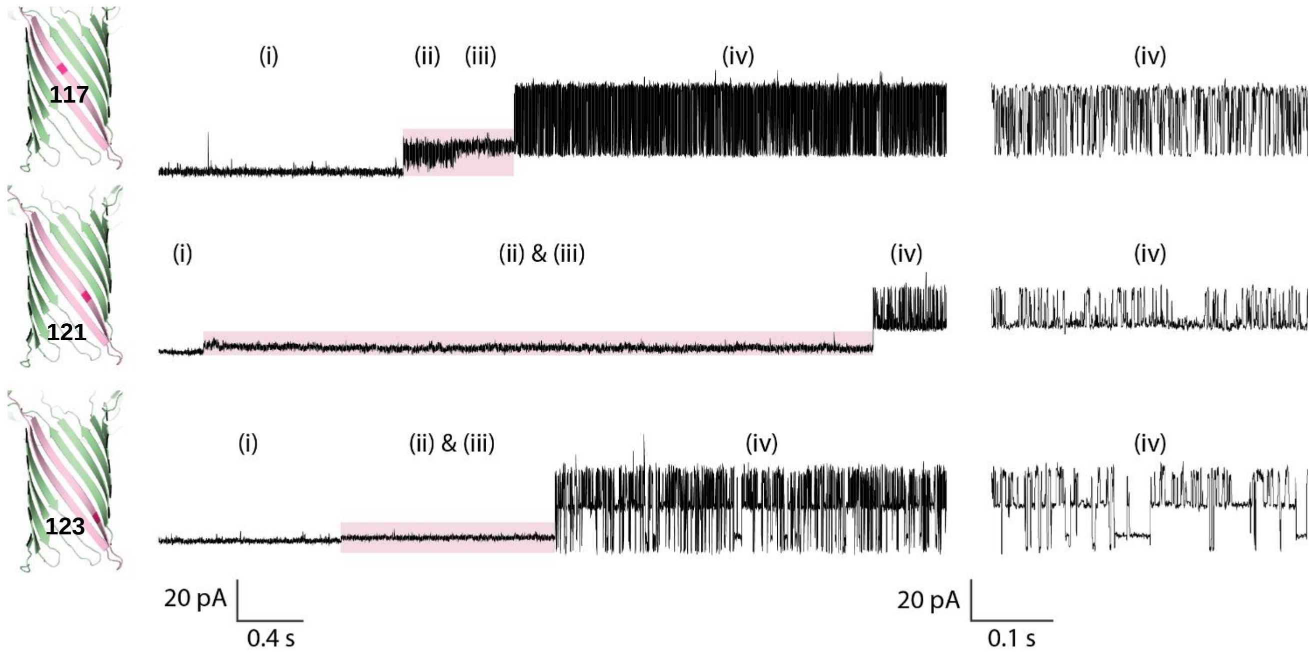

Figure S5. Single-channel recordings of Cys reactions with the CBT at sites 117, 121, and 123. Individual protein nanopores containing a cysteine at position 117, 121, or 123 (highlighted in pink) were modified in situ to generate CBT nanoreactors. In the reaction between the CBT(117) nanoreactor and Cys, two intermediates were resolved ($I_{res\%(i)} – I_{res\%(ii)}$ = 13.7%) (top trace). These levels could not be distinguished when Cys reacted with the CBT(121) nanoreactor ($I_{res\%(i)}$ – $I_{res\%(ii\&iii)}$ = 2.6%) (middle trace) or the CBT(123) nanoreactor ($I_{res\%(i)} – I_{res\%(ii\&iii)}$ = 1.7%) (bottom trace). The thiazoline product exhibited different current signatures at the three sites (right panel). Cys (5 mM) was added to the trans compartment. Current traces were filtered post-recording at 1000 Hz. Conditions: 2 M KCl, 20 mM HEPBS, pH 8.0, 20 µM EDTA, -50 mV, 20 ± 1 °C.

3.2 Direct attachment of the product molecule to a cysteine nanopore

To confirm that we formed the thiazoline product with CysOMe within the nanoreactor, we synthetized the corresponding amino-L-luciferin methyl ester (LucOMe) and derivatized it with p-maleimidophenyl isocyanate. The resultant LucOMe-Mal was used to modify the cysteine nanopore in situ to generate the proposed product directly (Figure S6). Given that the isocyanate was reactive towards both amine and carboxylate groups, derivatization of amino-L-luciferin—the product with Cys—with p-maleimidophenyl isocyanate was not straightforward and therefore not completed.

We added LucOMe-Mal to the trans compartment for direct attachment because the reaction with LucOMe-Mal was retarded when the molecule was added to the cis side, although it was less of a problem with CBT-Mal. It is therefore worth highlighting the dimensions of the product molecules (Figure S7). In the most extended form, LucOMe-Mal can elongate to ~2 nm (Figure S7), which is greater than the constriction of the β barrel (~1.4 nm).

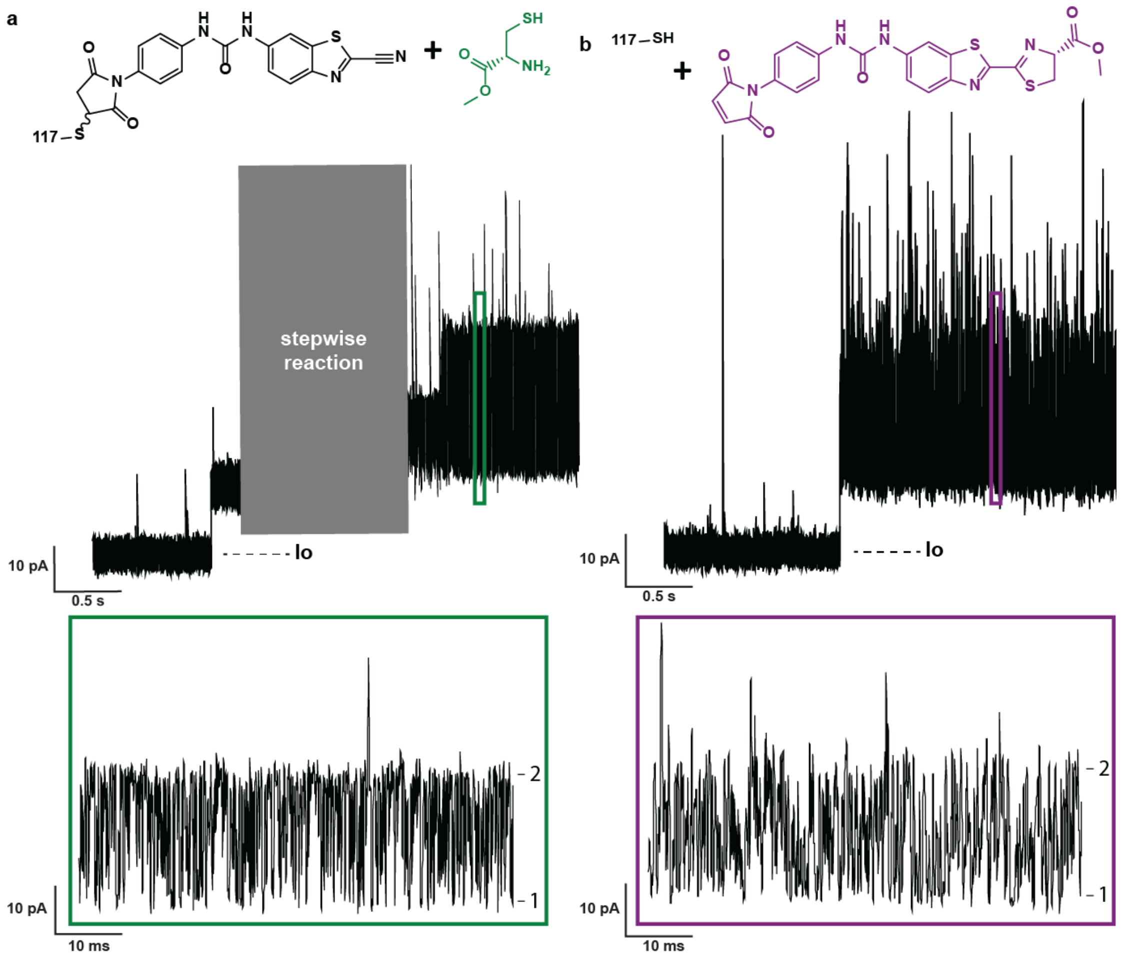

Figure S6. Direct attachment of the product molecule to a cysteine nanopore. a) Single-channel recordings showing the formation of the LucOMe product at Cys-117 by reaction of the CBT nanoreactor with CysOMe. After insertion of the cysteine nanopore, CBT-Mal was added as a freshly prepared DMSO solution (10 µM, cis; final 0.2% DMSO). After the step decrease in current occurred, indicating the generation of a CBT nanoreactor, CysOMe (5 mM) was added to the trans compartment. The product exhibited interconversions between two states (green box) with rates of transitions: $k_{1\text{-}2}$ = 11300 ± 100 s^{-1}; $k_{2\text{-}1}$ = 6120 ± 70 s^{-1}. **b)** Single-channel recordings showing the formation of the LucOMe product at Cys-117 by direct modification of the cysteine nanopore with LucOMe-Mal. LucOMe-Mal was added as a freshly prepared DMSO solution (10 µM, trans; final 0.2% DMSO). The product exhibited interconversions between two states (purple box) with rates of transitions: $k_{1\text{-}2}$ = 10700 ± 1000 s^{-1}; $k_{2\text{-}1}$ = 9380 ± 100 s^{-1}. No filter was applied post-recording to the current traces. Conditions: 2 M KCl, 20 mM HEPBS, pH 8.0, 20 µM EDTA, -50 mV, 20 ± 1 °C.

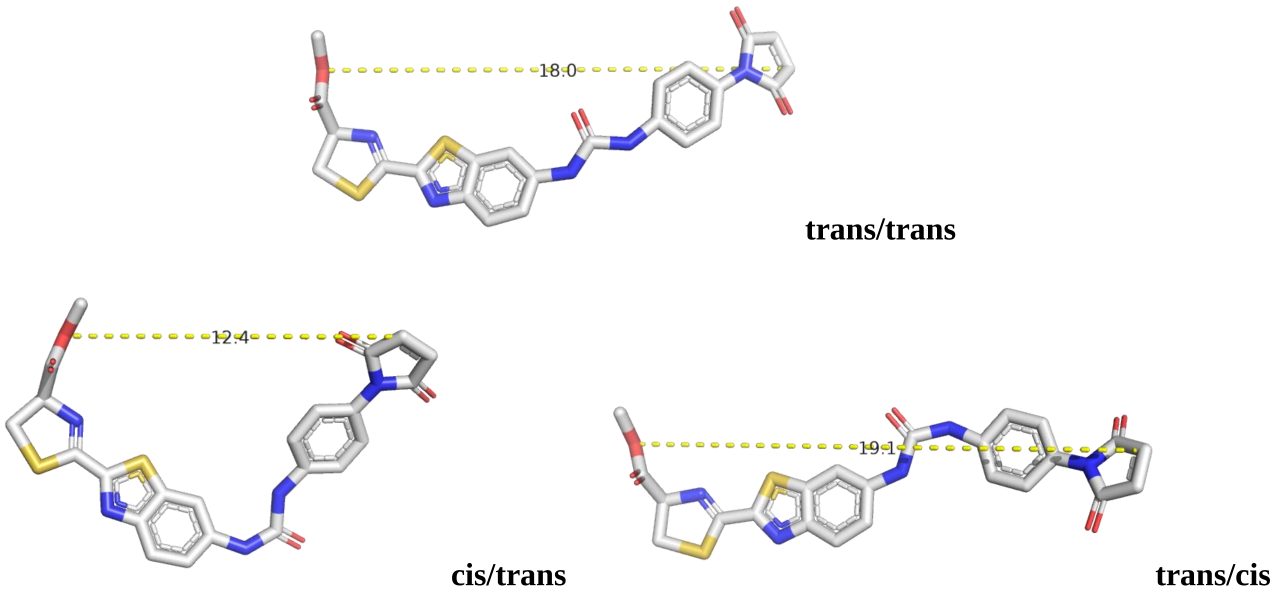

Figure S7. Structures and dimensions of LucOMe-Mal. The urea moiety was shown in the trans/trans, cis/trans, or trans/cis conformation. The cis/cis conformer was omitted as it is sterically unlikely. Dimensions are given in Å. The cis-trans isomerization in the urea group through rotation about the C−N bond has been reported to have an activation barrier of 11.3 kcal moL^{-1} in DMF/DMSO solutions[4] (>15000 s^{-1} at 20 °C).

3.4 Reversible thioimidate formation with hCys

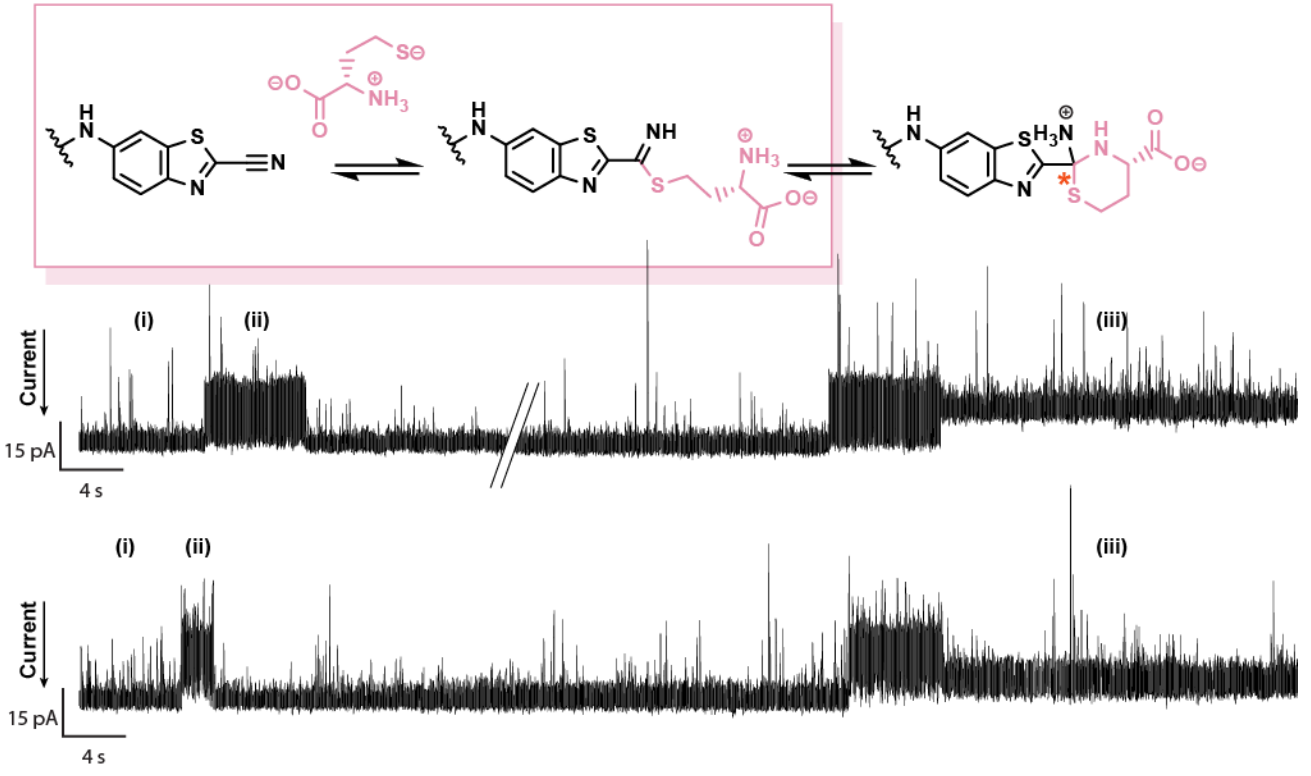

Figure S8. Reversible thioimidate formation with hCys. Single-channel recordings showing reversible thioimidate formation by hCys with two different CBT nanoreactors at -50mV. The three current levels correspond to the CBT nanoreactor (i), the thioimidate formed with hCys (ii), and the tetrahedral intermediate formed upon intramolecular cyclization (iii). The subsequent ammonia release in either case is not shown. A 200 Hz lowpass filter was applied post-recording. hCys (5 mM) was added to the trans compartment. Conditions: 2 M KCl, 20 mM HEPBS, pH 8.0, 20 µM EDTA, -50 mV, 20 ± 1 °C.

3.5 Ireversible condensation with NMeCys

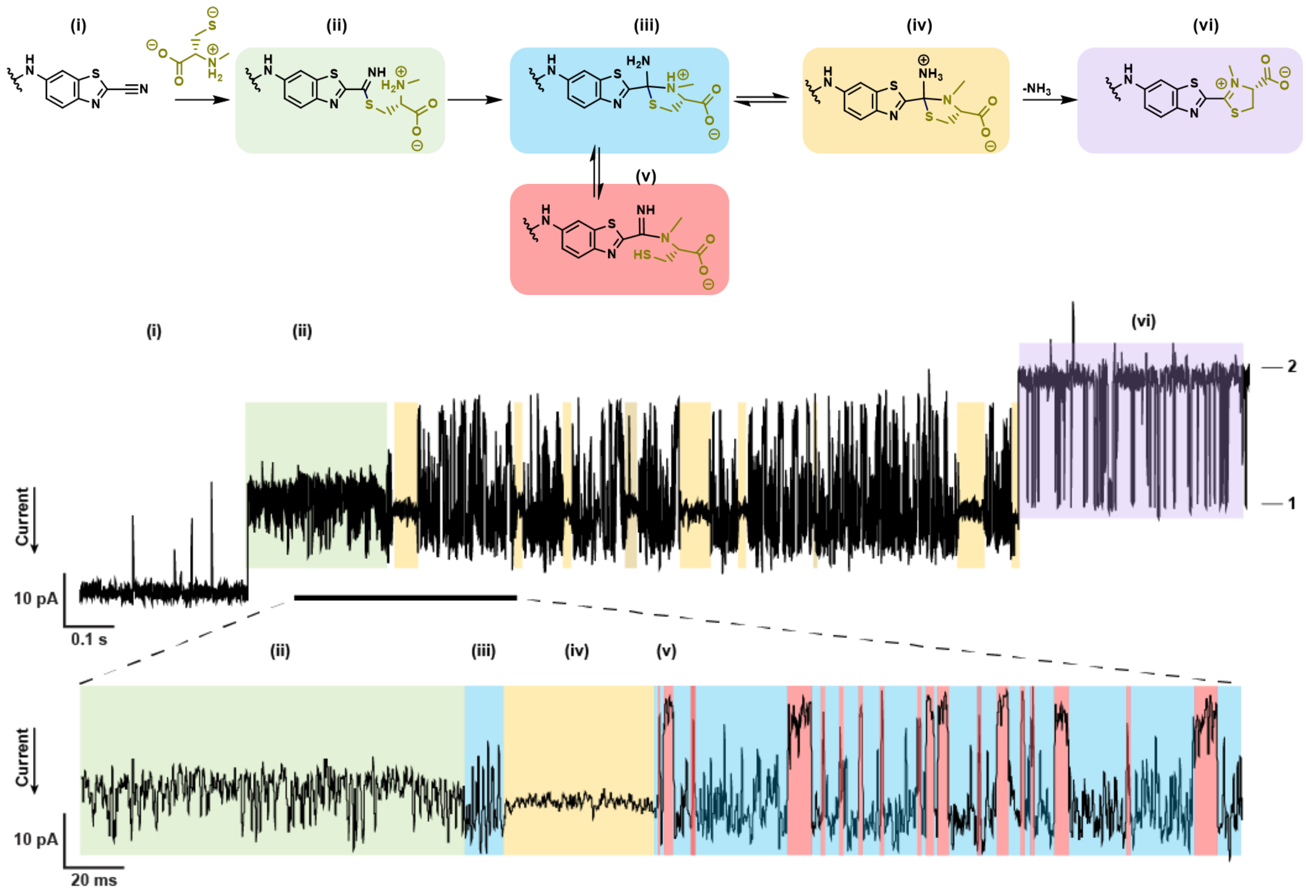

Figure S9. Single-channel recording of a reaction between NMeCys and a CBT nanoreactor. Upon reaction with CBT (i), four intermediate states were observed (ii-v). Based on the observations made with Cys, hCys, CysOMe, and Cys-Gly, we assigned the first intermediate state (ii) generated from CBT as the thioimidate; the last intermediate state (iv), which transitioned into the product (vi), we assigned as the tetrahedral intermediate with a protonated primary amine. We assigned state (iii) tentatively as the tetrahedral intermediate with a protonated tertiary amine, given that it was generated from the thioimidate (ii) and interconverted with both states (iv) and (v). We assigned the state (v) as the amidine, which only interconverted with state (iii). The ratio of I_{RMS} values for the states was I_{RMS} (I_i: I_{ii}: I_{iii}: I_{iv}: I_v) = 1.0: 2.0: 3.1: 1.0: 1.3. For the product level (vi), interconversions between two discrete current levels were seen (k_{1-2} = 742 ± 17 s^{-1}; k_{2-1} = 129 ± 3 s^{-1}). A 1000 Hz lowpass filter was applied post-recording. NMeCys (5 mM) was added to the trans compartment. Conditions: 2 M KCl, 20 mM HEPBS, pH 8.0, 20 µM EDTA, -50 mV, 20 ± 1 °C.

References

[1] Y. Qing, G. S. Pulcu, N. A. W. Bell, H. Bayley, *Angew. Chem. Int. Ed.* **2018**, *57*, 1218–1221.

[2] M. Montal, P. Mueller, *Proc. Natl. Acad. Sci. U. S. A.* **1972**, *69*, 3561–3566.

[3] F. Qin, A. Auerbach, F. Sachs, *Biophys. J.* **1996**, *70*, 264–280.

[4] P. Stilbs, S. Forsen, *J. Phys. Chem.* **1971**, *75*, 1901–1902.

[5] Y. J. Liu, W. H. Fang, *Sci. China, Ser. B Chem.* **2007**, *50*, 725–730.
